# A mouse model of lung cancer induced via intranasal injection for anticancer drug screening and evaluation of pathology

**DOI:** 10.1002/2211-5463.13486

**Published:** 2022-11-29

**Authors:** Ryo Tanaka, Shosei Yoshinouchi, Kento Karouji, Yuki Tanaka, Tsukasa Tominari, Michiko Hirata, Chiho Matsumoto, Yoshifumi Itoh, Chisato Miyaura, Masaki Inada

**Affiliations:** ^1^ Cooperative Major of Advanced Health Science Tokyo University of Agriculture and Technology Japan; ^2^ Testing and Research Laboratories HAMLI Co., Ltd. Ibaraki Japan; ^3^ Department of Biotechnology and Life Science Tokyo University of Agriculture and Technology Japan; ^4^ Institute of Global Innovation Research Tokyo University of Agriculture and Technology Japan; ^5^ Kennedy Institute of Rheumatology, Nuffield Department of Orthopaedics, Rheumatology and Musculoskeletal Sciences University of Oxford UK

**Keywords:** anticancer drug screening, cisplatin, intranasal injection, lewis lung carcinoma, NCI‐H460, orthotopic lung cancer model

## Abstract

The pathologies and lethality of lung cancers are associated with smoking, lifestyle, and genomic factors. Several experimental mouse models of lung cancer, including those induced via intrapulmonary injection and intratracheal injection, have been reported for evaluating the pharmacological effect of drugs. However, these models are not sufficient for evaluating the efficacy of drugs during screening, as these direct injection models ignore the native processes of cancer progression *in vivo*, resulting in the inadequate pathological formation of lung cancer. In the present study, we developed a novel intranasal injection model of lung cancer simulating the native lung cancer pathology for anticancer drug screening. A mouse lung cancer cell line (Lewis lung carcinoma; LCC) was intranasally injected into mouse lungs, and injected cell number‐dependent cancer proliferation was apparent in both the left and right lungs. Human non‐small‐cell lung cancer (NCI‐H460) cells were also intranasally injected into nude mice and similarly showed injected cell number‐dependent cancer growth. For the pharmacological evaluation of cisplatin, two different treatment frequencies were tested four times per month and twice a month. The intranasal injection model confirmed that cisplatin suppressed lung cancer progression to a greater extent under the more frequent treatment condition. In conclusion, these results indicated that our intranasal injection model is a powerful tool for investigating lung cancer pathology and may aid in the development of new anti‐lung cancer agents.

## Graphical abstract

We developed new mouse models of mouse and human lung cancers that were stably induced by intranasal injection. The intranasal injection model confirmed that cisplatin suppressed lung cancer progression, thus validating that this technique could be useful in investigating lung cancer pathologies and therapeutic agents.

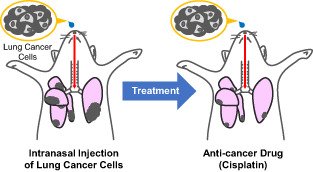

**Abbreviations**
CT: computed tomography
DHPN: N‐bis (2‐hydroxypropyl) nitrosamine
EGFR: epidermal growth factor receptor
GFP: green fluorescent protein
H&E: hematoxylin and eosin
IN: intranasal
IP: intraperitoneal
IPM: intrapulmonary
IT: intratracheal
IV: intravenous
LLC: lewis lung carcinoma
MRI: magnetic resonance imaging
NCI‐H460: human large‐cell lung cancer cell line
NNK: 4‐(methylnitrosamino)‐1‐(3‐pyridyl)‐1‐butanone
NSCLC: non‐small‐cell lung cancer
PD‐1: programmed cell death 1
PD‐L1: programmed cell death 1‐ligand 1
PDXs: patient‐derived xenografts
RFP: red fluorescent protein
SC: subcutaneous
SCLC: small‐cell lung cancer

Disease activities of lung cancer are a major cause of death in Japan, and the number of such patients has been increasing every year since 1981. Lung cancer is one of the leading causes of cancer‐related death worldwide, and the 5‐year survival rate of lung cancer is less than 30% in men and 50% in women [1]. Familial genetic factors and environmental factors, including smoking, air pollution, and radiation exposure, are reportedly risk factors for lung cancer [2].

Lung cancer is histologically classified into two main types, with about 15% of lung cancers being small‐cell lung cancer (SCLC) and about 85% non‐small‐cell lung cancer (NSCLC) [3]. NSCLC is further divided into three histologic subtypes: adenocarcinoma, squamous cell carcinoma, and large‐cell carcinoma [4, 5]; these subtypes account for 40%, 30%, and 15% of new lung cancer cases, respectively [6].

Mouse models of lung cancer consist of three major types: spontaneous models, drug‐induced models, and lung cancer cell‐injected models. A/J mice are widely used as a spontaneous model associated with developing pulmonary cancer with age [7]. Drug‐induced models of lung cancer have been developed using carcinogens, such as 4‐(methylnitrosamino)‐1‐(3‐pyridyl)‐1‐butanone (NNK), N‐bis (2‐hydroxypropyl) nitrosamine (DHPN) and urethane; however, it takes at least 16 weeks after the administration of these agents for lung cancer to form [8, 9, 10].

Two types of mouse models of lung cancer induced via injection have been developed: a xenograft (heterogeneous) model and a syngeneic (homogeneous) model. The xenograft model is induced by injecting human lung cancer cells into immunodeficient mice [11]. By contrast, the syngeneic model is prepared by injecting several established mouse lung cancer cells [12]. In patient‐derived xenografts (PDXs), immunodeficient mice are transplanted with patient‐derived cancer explants; this approach is widely used to conduct cancer research in subcutaneous (SC) injection models [13]. In the SC model, the microenvironment of tissues differs for each different type of organ‐originating primary cancer, thus making it difficult to evaluate the drug efficacy for various organ‐derived cancers using only the model of SC injection [5, 10]. Other evaluation models of lung cancer in the mouse, such as intravenous (IV) injection models, are commonly used in studies of cancer metastasis; therefore, such models are not suitable for evaluating the proliferation of lung‐originated primary lung cancer [14].

Given the above, recent studies have reported the development of orthotopic models via the direct injection of lung cancer cells into the lung or trachea. In intrapulmonary (IPM) injection, the skin over the left chest is incised, and cancer cells are directly injected into the left lung parenchyma [15]. In lung cancer models induced via intratracheal (IT) injection, lung cancer cells are injected into the trachea using a tube, probe, or needle to seed cancer into both the left and right lungs or right posterior lobe [16, 17]. These IPM and IT injection models are highly invasive for mice, and cancer is often formed outside the lung as a consequence of dissemination during injection. Therefore, a novel model of lung cancer is needed for cancer drug screening.

In the present study, we newly developed an intranasal (IN) injection model of lung cancer cells based on mouse infectious disease models, such as that involving the influenza virus.

## Materials and methods

### Animals and reagents

C57BL/6J and BALB/c‐nu mice (5‐ to 8‐week‐old, males) were obtained from Charles River Laboratories Japan, Inc. (Kanagawa, Japan). All animal studies were conducted in accordance with Guidelines for Care and Use of Laboratory Animals and the act on animal welfare in Testing and Research Laboratories, (HAMLI Co., LTD., Ibaraki, Japan, Approved ID: No. 17‐112). The mice were allowed *ad libitum* access to water and food pellets (CRF‐1, MF; Oriental Yeast Co., LTD., Tokyo, Japan) and housed in polysulfone cages (170W × 280D × 100H mm, Allentown Caging Equipment Co., Allentown, NJ, USA) at room temperature (24 ± 3 °C) and 50 ± 20% humidity under a 12‐h light–dark cycle (light from 7:00 am to 7:00 pm).

The Lewis lung carcinoma (LLC) murine lung cancer cell line was obtained from the Japanese Collection of Research Bioresources Cell Bank (Osaka, Japan). A human large‐cell lung cancer cell line (NCI‐H460) was obtained from the American Type Culture Collection (VA, USA). These cells were cultured in RPMI‐1640 Medium supplemented with 10% fetal bovine serum (FBS; Cytiva, Tokyo, Japan), 100 U·mL^−1^ of penicillin (FUJIFILM Wako Pure Chemical Corp., Osaka, Japan), and 100 μg·mL^−1^ of streptomycin (FUJIFILM Wako Pure Chemical Corp.) at 37 °C under 5% CO_2_ in air.

### Mouse models of lung cancer

Sub‐confluent cultures of LLC cells were harvested using 0.05% trypsin–EDTA (Thermo Fisher Scientific Inc., Waltham, MA, USA), washed in phosphate‐buffered saline (PBS; FUJIFILM Wako Pure Chemical Corp.), and resuspended as single‐cell suspensions in PBS at concentrations of 1 × 10^6^, 1 × 10^7^, 5 × 10^7^ or 1 × 10^8^ cells·mL^−1^. NCI‐H460 cells were suspended in PBS at 5 × 10^7^ cells·mL^−1^.

For the IPM injection, mice were anesthetized via intraperitoneal (IP) administration with three types of mixed anesthetic agents: medetomidine (Nippon Zenyaku Kogyo Co., Ltd., Fukushima, Japan), midazolam (Astellas Pharma Inc., Tokyo, Japan) and butorphanol (Meiji Seika Pharma Co., Ltd., Tokyo, Japan). The skin on the left side of the chest was incised by 1 cm, and the soft tissue was dissected in order to expose the thoracic ribs and intercostal space [15]. LLC cells (1 × 10^6^) suspended in 100 μL of PBS were injected into the left lung parenchyma using a 29‐G syringe needle (Terumo Corp., Tokyo, Japan). The incised site was sutured, and the mice were allowed time to recover under a warm lamp until fully awake.

For the IT injection, mice were anesthetized with three types of mixed anesthetic agents by IP administration. A surgical skin incision of the neck was performed to expose the trachea. LLC cells (1 × 10^6^) suspended in 100 μL of PBS were injected into the main bronchi using a 29‐G syringe needle [18]. The incised site was sutured, and the mice were allowed time to recover under a warm lamp until fully awake.

For the IN injection, we modified the established infection models for influenza virus and rhinovirus [19, 20, 21]. Mice were anesthetized with 3% isoflurane (Mylan Inc., Canonsburg, PA, USA) by inhalation. LLC cells (1 × 10^5^, 1 × 10^6^, or 1 × 10^7^) or NCI‐H460 cells (5 × 10^6^) suspended in 100 μL of PBS were then intranasally injected.

The body weight of mice was measured at the indicated time points. The survival of 5–12 mice was monitored during the experiments with reference to a previous study [22].

### Cisplatin administration

LLC cells (1 × 10^7^) were intranasally injected on day 0 as described above. Cisplatin (5 mg·kg^−1^; Bristol Myers Squibb Company, New York, NY, USA) was intravenously administered to mice on days 1 and 8. NCI‐H460 cells (5 × 10^6^) were intranasally injected on day 0, and cisplatin was intravenously administered to mice on days 1 and 8 (Case 1) or on days 1, 8, 15, and 22 (Case 2). After 3 or 4 weeks, mice were sacrificed, and lungs were collected in 10% neutral‐buffered formalin for histopathology.

### Histopathology staining and analyses

Lungs fixed with 10% neutral‐buffered formalin were embedded in paraffin and cut into 5‐μm sections using a microtome (Leica Biosystems, Wetzlar, Germany). The sections were stained with hematoxylin (FUJIFILM Wako Pure Chemical Corp.) and eosin (Sakura Fineteck Japan Co., Ltd., Tokyo, Japan) (H&E) or May‐Grunwald staining solution (Merck KGaA, Darmstadt, Germany) and Giemsa staining solution (Merck KGaA) and analyzed for the lung area and cancer area using WinROOF2015 (MITANI Corporation, Fukui, Japan). The cancer area in the lung was calculated as the lung cancer area ratio (%) = lung cancer area/lung area × 100.

### Statistical analyses

Data were presented as the mean ± standard deviation (SD). The significance of differences between two groups was analyzed using the Dunnett's test or log‐rank test for Kaplan–Meier survival curves (BellCurve for Excel; Social Survey Research Information Co., Ltd., Tokyo, Japan).

## Results

### The comparison of the mouse lung cancer growth in the IPM, IT, and IN injected models

**Fig. 1:**
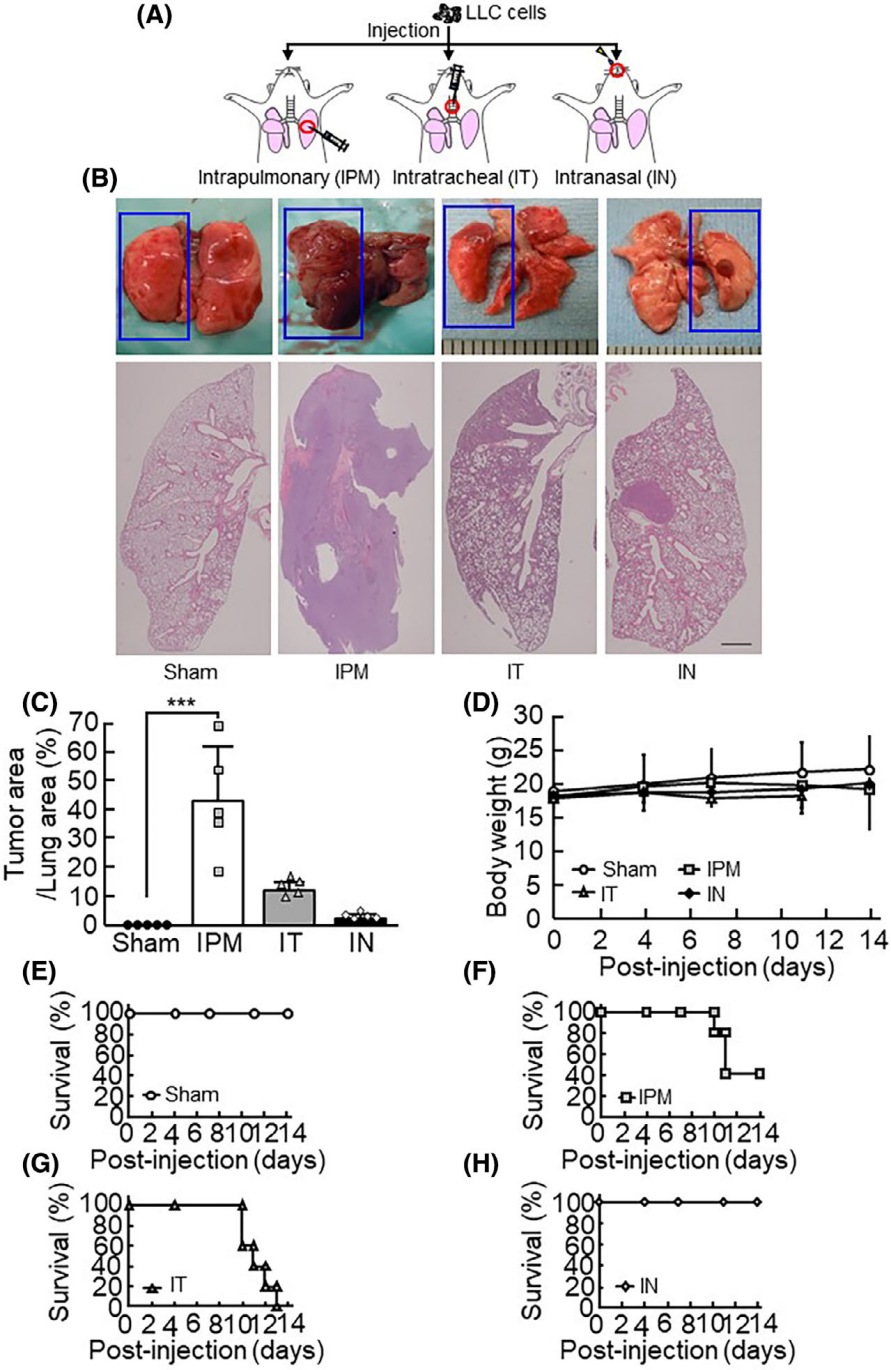
The comparison of the orthotopic lung cancer models. (A) A schematic presentation concerning the establishment of the orthotopic models. Red circles indicate the injection site (*n* = 5 per group). (B) Representative images of lung sections stained with H&E in each mouse model. The ruler 1 scale bar indicates 1 mm. (C) The cancer area per lung area was calculated. The data are expressed as the mean ± SD of 5 mice per group. (D) The body weight of mice was measured at the indicated time points. The data are expressed as the mean ± SD of 5 mice per group. (E–H) Survival curves of each mouse model during the experiment (*n* = 5 per group). A significant difference between the two groups was noted: ***P < 0.001 (Dunnett's test).

We examined the differences in the phenotypes of the lung cancer models for each injection method. Three routes IPM, IT, and IN injection are illustrated in Fig. 1A. Mouse LLC cells (1 × 10^6^) were injected via IPM, IT, and IN injection.

The cancer formation was observed by H&E staining in the left lobe and thoracic cavity in the IPM, IT, and IN model mice (Fig. 1B). IPM and IT injection induced lung cancer growth in the entire left lobe and thoracic cavity broadly, but IN injection showed limited growth in the thoracic cavity (Fig. 1B,C). The body weight in these models was monitored and compared with that of sham animals; there were no significant differences between these groups (Fig. 1D). The survival rates of the IPM, IT, and IN injection mice were examined. The IPM and IT model mice showed lethality from 10 to 14 days after injection (Fig. 1E–G), while all IN model mice survived (Fig. 1H).

### Various numbers of injected mouse cancer cells in the IN injected model

**Fig. 2:**
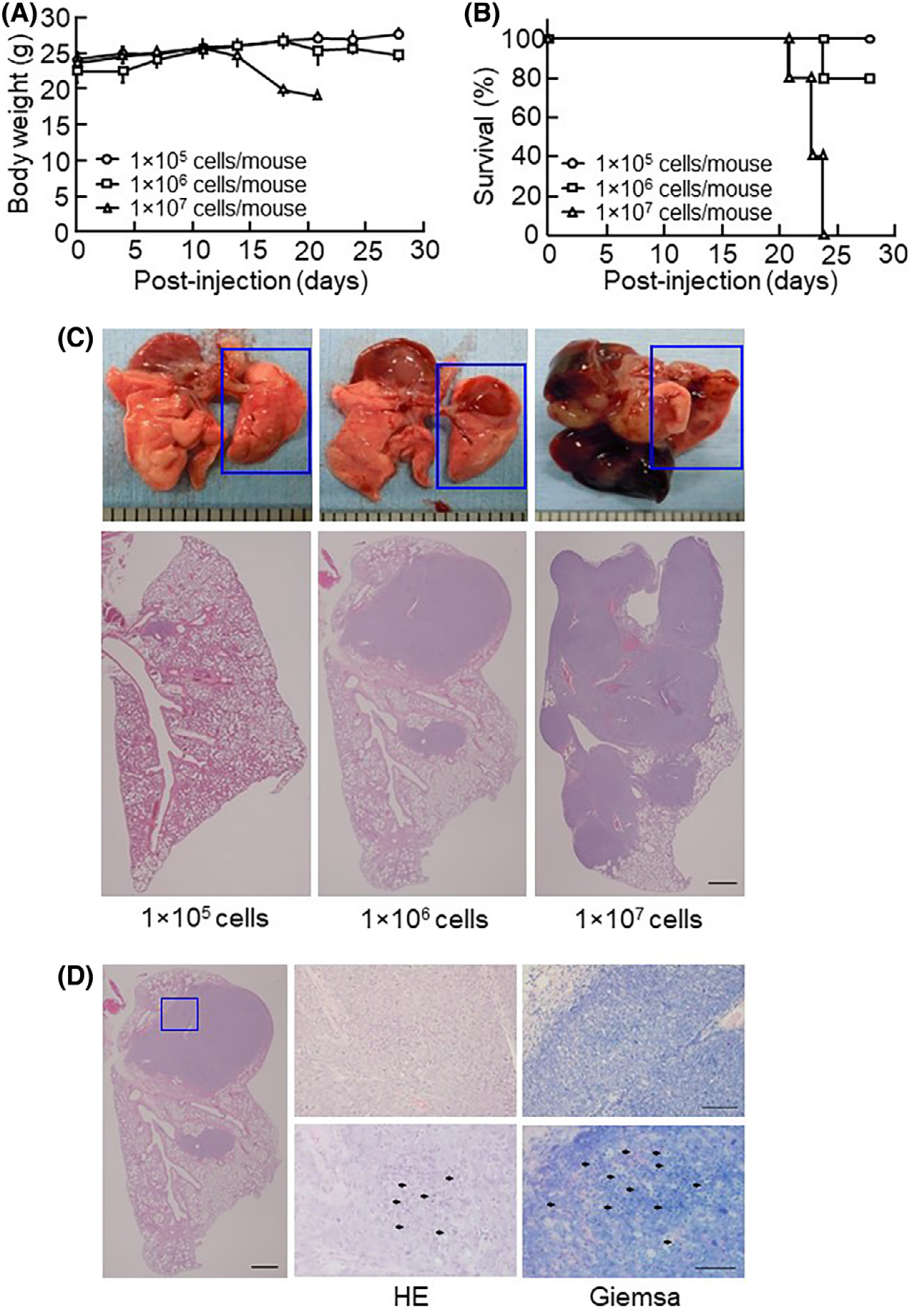
The injection of LLC cells dose‐dependently induced lung cancer formation in mice. (A) The body weight of mice was measured at the indicated timepoint. The data are expressed as the mean ± SD of 5 mice per group. (B) Survival curves of mice injected with LLC cells (*n* = 5 per group). (C) Representative images of lung and histological lung sections stained with H&E in mice injected with LLC cells. The ruler 1 scale bar indicates 1 mm. (D) Giemsa‐stained lung sections in mice injected with 1 × 10^6^ LLC cells. A magnified view of the boxed area of the image of the HE and Giemsa‐stained section is shown in the left panel. Scale bars indicate 100 μm (left image) and 50 μm (middle and right images). Black arrows show infiltrating leukocytes.

We examined the influence of the number of injected LLC cells on cancer growth in IN injection mice (Fig. 2). The body weight was decreased in 1 × 10^7^ LLC cell‐injected mice on day 14 and in 1 × 10^6^ LLC cell‐injected mice on day 21, with no changes noted in 1 × 10^5^ LLC cell‐injected mice (Fig. 2A). The lethal ratio was increased on day 21 in 1 × 10^7^ LLC cell‐injected mice and on day 24 in 1 × 10^6^ LLC cell‐injected mice, while all mice injected with 1 × 10^5^ LLC cells survived (Fig. 2B). Regarding anatomical observations, the proliferating area of lung cancer increased depending on the injected cell dose, such as by increasing the injected number of LLC cells in the IN injection model. Regarding the histopathology, H&E staining revealed lung cancer formation in the hilum of both the left and right lungs, and proliferating lesions increased depending on the number of injected LLC cells (Fig. 2C). We performed Giemsa staining to investigate lymphocyte infiltration in the area surrounding lung cancer tissue. This indicated that leukocytes were recruited by the area surrounding the lung cancer tissue (Fig. 2D), and the histological pattern corresponded with clinical cases of human lung cancer [23, 24].

### Cisplatin efficacy in the IN injection model using mouse lung cancer cells

**Fig. 3:**
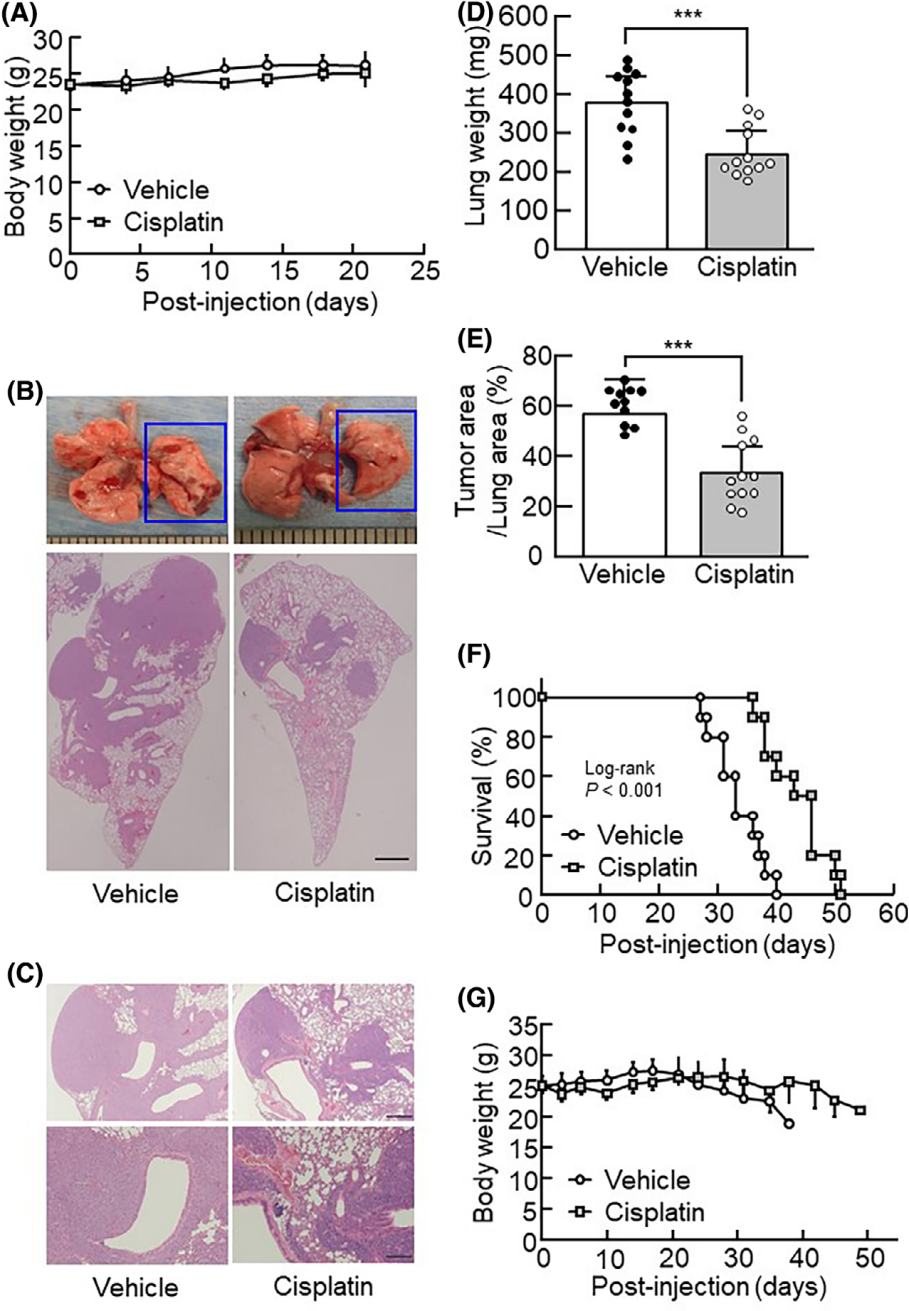
Therapeutic effects of cisplatin on an IN injection model with LLC cells. (A) The body weight of mice was measured at the indicated timepoint (short period). The data are expressed as the mean ± SD of 12 mice per group. (B) Representative images of histological lung sections stained with H&E in IN injection model mice treated with cisplatin. The ruler 1 scale bar indicates 1 mm. (C) Upper images show highly magnified images of the box indicated area in the H&E images of (B). Lower images show highly magnified views of the boxed areas in the upper images. The scale bars in the upper and lower images indicate 500 and 200 μm, respectively. (D) The lung weight was measured on day 21. The data are expressed as the mean ± SD of 12 mice per group. (E) The cancer area per lung area was calculated on day 21. The data are expressed as the mean ± SD of 12 mice per group. (F) Survival curves of IN injection model mice treated with cisplatin. (*n* = 10 per group). The log‐rank test indicated a significant difference between the two groups; *P* < 0.001. (G) The body weight of mice was measured at the indicated timepoint (long period). The data are expressed as the mean ± SD of 10 mice per group. A significant difference between the two groups was noted using the Dunnett's test; ***P < 0.001.

We examined the effects of the anticancer drug cisplatin on the IN injection model using mouse lung cancer cells. Cisplatin was intravenously administered twice (once a week) to mice that had received IN injection with 1 × 10^7^ LLC cells. As a result, cisplatin treatment showed no effect on the body weight (Fig. 3A). Regarding the histopathological findings using H&E staining, vehicles showed an increased area of lung cancer proliferation, whereas cisplatin‐treated mice showed a decreased area (Fig. 3B,C). Cisplatin treatment also significantly decreased both the lung weight and cancer area on day 21 (Fig. 3D,E) and increased the survival ratio (lethality at 40 days for vehicle vs. 50 days for cisplatin treatment; Fig. 3F). Cisplatin treatment also induced the maintenance of consistent body weight for 50 days (Fig. 3G).

### Cisplatin efficacy in the IN injection model using human lung cancer cells

**Fig. 4:**
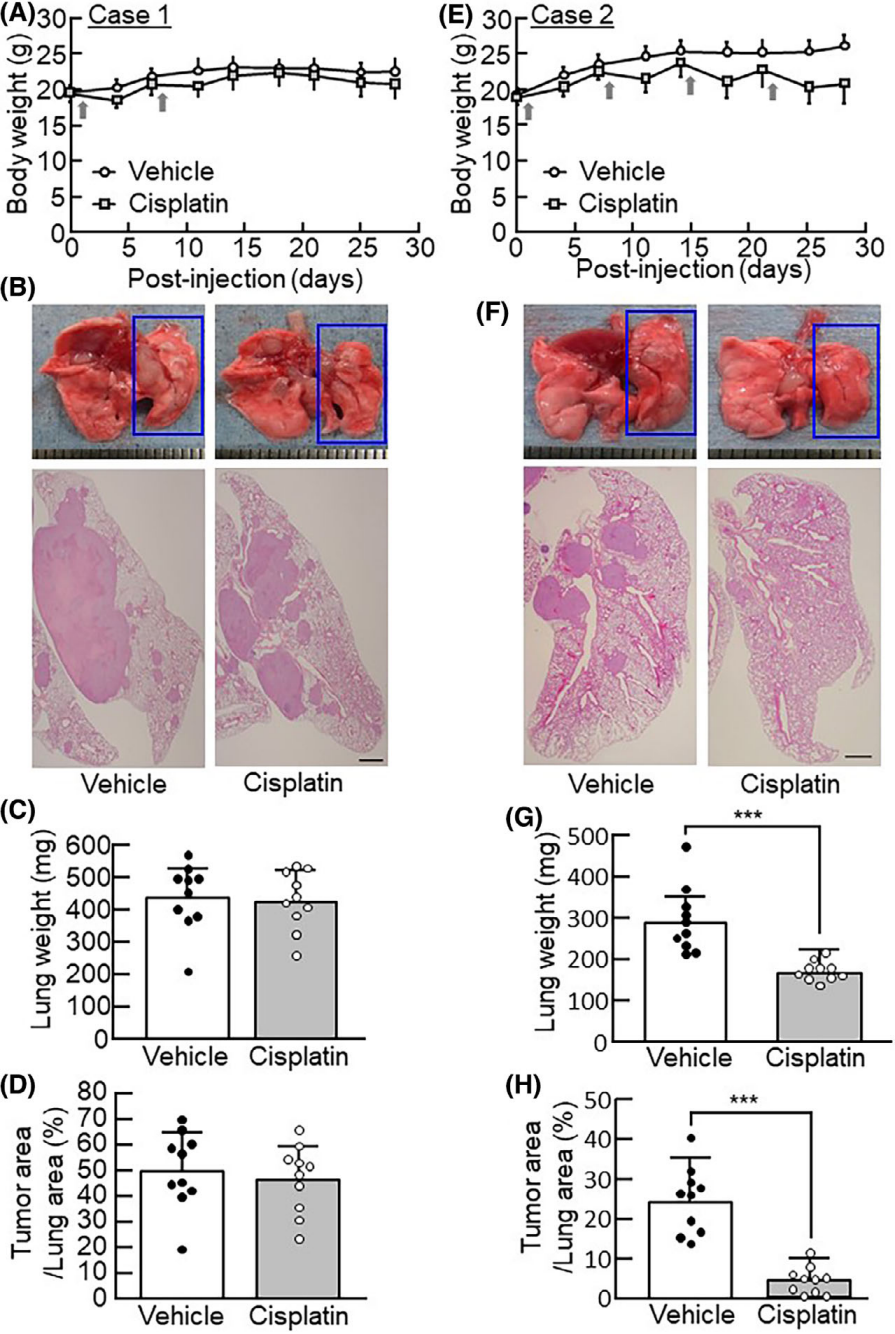
Therapeutic effects of cisplatin on an IN injection model with NCI‐H460 cells. (A) The body weight of mice was measured at the indicated timepoint in mice administered cisplatin twice (day 1 and 8: Case 1). Gray arrows indicate days of cisplatin administration. The data are expressed as the mean ± SD of 10 mice per group. (B) Representative images of lung histological sections stained with H&E in mice administered cisplatin twice on day 28. The ruler 1 scale bar indicates 1 mm. (C) The lung weight of mice administered cisplatin twice was measured on day 28. The data are expressed as the mean ± SD of 10 mice per group. (D) The cancer area per lung area of mice administered cisplatin twice was calculated on day 28. The data are expressed as the mean ± SD of 10 mice per group. (E) The body weight of mice was measured at the indicated timepoint in mice administered cisplatin four times (day 1, 8, 15, and 22: Case 2). Gray arrows indicate days of cisplatin administration. The data are expressed as the mean ± SD of 10 mice per group. (F) Representative images of lung histological sections stained with H&E in mice administered cisplatin four times on day 28. The ruler 1 scale bar indicates 1 mm. (G) The lung weight of mice administered cisplatin four times was measured on day 28. The data are expressed as the mean ± SD of 10 mice per group. (H) The cancer area per lung area of mice administered cisplatin four times was calculated on day 28. The data are expressed as the mean ± SD of 10 mice per group. A significant difference between the two groups was noted using the Dunnett's test; ***P < 0.001.

We examined the effects of the anticancer drug cisplatin on the IN injection model using NCI‐H460 cells. We examined the effect of two different timings of cisplatin administration for the NCI‐H460 IN injection model. First, cisplatin was administered twice (days 1 and 8; Case 1). Case 1 showed that cisplatin treatment was not effective, and neither the lung weight nor the lung cancer area was significantly decreased by twice‐monthly treatment with cisplatin (Fig. 4A–D). In a second experiment, mice were administered cisplatin four times (days 1, 8, 15, and 22; Case 2). Case 2 showed that lung cancer proliferation around the bronchi was significantly suppressed by the four‐time administration of cisplatin (Fig. 4E,F), and the lung weight and cancer area were also remarkably decreased on day 28 in this experiment (Fig. 4G,H).

## Discussion

In the present study, we developed a new model of lung cancer induced via IN injection. This IN injection model was prepared by injecting mouse or human lung cancer cells into mice via the nasal cavity under mild anesthesia without any surgical operations. Other models, such as IPM and IT injection models, require experimental techniques involving needle insertion and carry a risk of inducing cancer outside the lung via dissemination during injection. The IN injection model thus has the benefits of being less invasive and more easily prepared than other models for drug screening.

This IN injection method has been commonly used for establishing infectious models of influenza virus and rhinovirus [19, 20, 21]. In IN injection models, these viruses reach the alveoli via spontaneous inhalation by mice. Since the alveoli are the sites of the initial development of lung cancer, injected lung cancer cells reach the alveoli along a similar pathway to the influenza virus and rhinovirus [25, 26]. McGee et al. performed H&E staining of mouse lung tissue infected with influenza virus. The infected lesion expanded from the bronchiole to the alveoli, indicating that intranasally injected viruses reach the alveoli through the bronchiole. Correspondingly, our IN injection model showed that lung cancer primarily forms tumors around bronchioles and alveoli, as shown in Figs 1B and 2C. Thus, the pathogenesis of IN injected lung cancer was shown to closely resemble the pathogenesis of lung‐originating primary lung cancer, indicating that the IN injection model is useful for observing lung‐originating lung primary cancers and also for evaluating anti‐lung cancer drug efficacy.

Recent studies have demonstrated various diagnostic methods of cancer mouse models, using computed tomography (CT), magnetic resonance imaging (MRI), and biofluorescence/luminescence imaging with a green fluorescent protein (GFP), red fluorescent protein (RFP), or luciferase‐expressing cancer cells. Liu et al. [27] established an orthotopic mouse model of lung cancer via intrathoracic injection and performed a time course analysis using spiral CT. CT has advantages over other modalities in noninvasive live imaging and is useful for imaging hard tissues, including bone. Since lung cancer frequently metastasizes to bone, CT is a powerful tool for evaluating lung cancer bone metastasis. MRI has advantages in the live monitoring of cancer progression. Partecke et al. [28] performed 7‐Tesla MRI of orthotopic pancreatic cancer models and noted time‐dependent cancer growth and liver metastasis. Seven‐Tesla MRI generates super‐high‐resolution images of the lung, suggesting that MRI is useful for precisely evaluating the cancer volume in experimental models of lung cancer. Other medical imaging models, such as those involving bio‐fluorescence/luminescence imaging, are also useful for evaluating cancer progression and screening anticancer drugs. Kuchimaru et al. [29] successfully visualized the time course of cancer progression using luciferase‐expressing cancer cells. Bio‐fluorescence/luminescence can detect even single cells, and the intensity of the fluorescence/luminescence depends on the number of cells; therefore, bio‐fluorescence/luminescence imaging precisely reflects cancer progression. Given the above advantages, these imaging techniques are widely used in cancer research and are useful for monitoring cancer progression without sacrificing animals. These imaging techniques enable the time course of cancer progression to be analyzed on a long‐term basis in live animals. Further studies will be needed to analyze the phenotypes of IN injection models using these noninvasive imaging techniques for the precise evaluation of cancer progression.

Cisplatin has been reported to exert anticancer effects on lung cancer [30] and is frequently used to treat lung cancer clinically. Recent studies have described the development of several anti‐lung cancer drugs, including afatinib, irinotecan, and paclitaxel [11, 31, 32]. Afatinib irreversibly binds to epidermal growth factor receptor (EGFR) and Her2 and inhibits tyrosine kinase followed by the activation of the Ras‐MAPK and PI3K‐Akt signaling pathway, which play critical roles in the cancer cell proliferation, migration, and survival. Li et al. [31] reported that afatinib remarkably suppressed cancer growth, using xenograft and transgenic models of lung cancer. Irinotecan is a topoisomerase I inhibitor and disrupts DNA replication in cancer cells, leading to cancer regression. Kunimoto et al. [32] reported strong cancer‐inhibitory effects of 7‐ethyl‐10‐hydroxy‐camptothecin, the active metabolite of irinotecan, using an SC mouse model of LLC. Paclitaxel promotes tubulin polymerization and stabilizes the microtubule to inhibit nuclear division. Yamori et al. [11] demonstrated the cancer‐inhibitory effects of paclitaxel using an SC xenograft model of human lung cancer.

A recent target of anti‐lung cancer treatment has been immune checkpoint inhibitors, including inhibitors of programmed cell death 1 (PD‐1) and programmed cell death 1‐ligand 1 (PD‐L1). PD‐1 and PD‐L1 are immune checkpoint molecules and induce immune suppression, leading to cancer progression. Thus, their inhibitors are used as anticancer drugs for lung cancer. The drug efficacy of anti‐PD‐1 and anti‐PD‐L1 antibodies has been previously reported using an IPM injection model [33]. Our IN injection model has potential utility for the precise evaluation of drug efficacy; therefore, we intend to investigate other anticancer agents, such as anticancer antibodies, using our IN injection model.

In conclusion, we newly developed an IN injection model of lung cancer that is easily prepared via spontaneous inhalation in mice. Cancer progression can also be controlled with our IN injection model by changing the number of injected lung cancer cells. Once the injected cells reach the alveoli as appropriate, where lung cancer originates, they form primary lung cancer lesions. The IN injection model can also be applied to various gene‐deficient mice to investigate the roles of targeted genes in lung cancer pathology. These results indicate that our newly developed IN injection model is a powerful tool for investigating lung cancer pathology and developing new therapeutic agents for lung cancer.

## Conflict of interest

The authors declare no conflict of interest.

## Author contributions

RT, CMi, and MI conceived of and designed the experiments; RT, SY, KK, and YT acquired the data; SY, TT, MH, CMa, and YI analyzed and interpreted the data; RT, SY, YI, CMi, and MI wrote the manuscript; all authors reviewed the manuscript.

## Data Availability

Data are available from the corresponding author upon reasonable request.
